# Tea and coffee consumption in relation to vitamin D and calcium levels in Saudi adolescents

**DOI:** 10.1186/1475-2891-11-56

**Published:** 2012-08-20

**Authors:** Abdulaziz Al-Othman, Sara Al-Musharaf, Nasser M Al-Daghri, Sobhy Yakout, Khalid M Alkharfy, Yousef Al-Saleh, Omar S Al-Attas, Majed S Alokail, Osama Moharram, Shaun Sabico, Sudhesh Kumar, George P Chrousos

**Affiliations:** 1 Prince Mutaib Chair for Biomarkers of Osteoporosis, King Saud University, Riyadh, KSA; 2 College of Applied Medical Sciences, King Saud University, Riyadh, KSA; 3 College of Science, King Saud University Women's Section, Riyadh, KSA; 4 Biomarkers Research Program, Biochemistry Department, College of Science, King Saud University, Riyadh, Kingdom of Saudi Arabia (KSA; 5 Center of Excellence in Biotechnology Research, King Saud University, Riyadh, KSA; 6 Clinical Pharmacy Department, College of Pharmacy, King Saud University, Riyadh, KSA; 7 College of Medicine, King Saud University of Health Sciences, Riyadh, KSA; 8 King Abdulaziz University Hospital, King Saud University, Riyadh, KSA; 9 Clinical Sciences Research Institute, Diabetes and Metabolism Unit, Warwick University, Coventry, CV47AL, UK; 10 First Department of Pediatrics, Athens University Medical School, Athens, 11527, Greece; 11 Prince Mutaib Bin Abdullah Chair for Osteoporosis, Biochemistry Department, College of Science, King Saud University, PO Box, 2455, Riyadh, 11451, Kingdom of Saudi Arabia

**Keywords:** Coffee intake, Tea intake, Vitamin D levels, Saudi adolescents

## Abstract

**Background:**

Coffee and tea consumption was hypothesized to interact with variants of vitamin D-receptor polymorphisms, but limited evidence exists. Here we determine for the first time whether increased coffee and tea consumption affects circulating levels of 25-hydroxyvitamin D in a cohort of Saudi adolescents.

**Methods:**

A total of 330 randomly selected Saudi adolescents were included. Anthropometrics were recorded and fasting blood samples were analyzed for routine analysis of fasting glucose, lipid levels, calcium, albumin and phosphorous. Frequency of coffee and tea intake was noted. 25-hydroxyvitamin D levels were measured using enzyme-linked immunosorbent assays.

**Results:**

Improved lipid profiles were observed in both boys and girls, as demonstrated by increased levels of HDL-cholesterol, even after controlling for age and BMI, among those consuming 9–12 cups of coffee/week. Vitamin D levels were significantly highest among those consuming 9–12 cups of tea/week in all subjects (p-value 0.009) independent of age, gender, BMI, physical activity and sun exposure.

**Conclusion:**

This study suggests a link between tea consumption and vitamin D levels in a cohort of Saudi adolescents, independent of age, BMI, gender, physical activity and sun exposure. These findings should be confirmed prospectively.

## Background

Coffee is arguably the most widely consumed non-carbonated beverage around the world [1]. In Saudi Arabia, the Arabian coffee “Gahwa”, is a common hot drink. It is a mainstay drink served to guests, consumed almost daily in most Saudi homes, and is served heavily in all local social occasions and gatherings. This coffee is served in traditional standard small cups (around 30 ml each) and is usually consumed with fresh or preserved dates.

Traditionally, the beneficial effects of coffee have been attributed solely to caffeine, but increasing evidence suggests that other compounds may contribute to the health benefits of coffee consumption [2]. In fact, coffee is a complex mixture of compounds composed of more than a thousand different chemicals, including carbohydrates, lipids, nitrogenous compounds, vitamins, minerals, alkaloids and phenolic compounds among them caffeine, cafestol, and kahweol [3].

The effects of caffeine consumption in children is limited, and most studies have focused on behavioral effects [4]. In general, caffeine doses less than 3.0 mg/kg of body weight have not resulted in any adverse effects on children in controlled clinical trials, but concerns regarding its effects on the developing nervous system have led to recommendations that daily intake should be limited to 2.5 mg/kg of body weight [5].

With regards to bone metabolism, increased coffee consumption was documented to cause a negative shift in overall calcium balance, but was not related to bone turnover in postmenopausal women with fracture osteoporosis [6]. Furthermore, caffeine was observed to interact with the TT genetic variant of vitamin D receptor [7]. Hannan and colleagues however reported no association between caffeine intake on bone metabolism [8]. There is scarcity of information with regards to tea consumption and vitamin D, although in terms of skeletal effect, it has been reported that excessive consumption of brewed tea can lead to skeletal fluorosis and increased bone mineral density [9]. This study aims to determine associations between the frequency of coffee and tea consumption and serum measures of vitamin D and calcium levels in a cohort of adolescent Saudis.

## Methods

In this cross-sectional study, a total of 330 (155) Saudi boys and (175) girls aged 11–14 years were randomly selected from the existing Biomarkers Screening in Riyadh Program (RIYADH Cohort), a capital-wide study composed of randomly selected individuals from different Primary Health Care Centers (PHCCs) in Riyadh, KSA. The study was carried out at the Biomarkers Research Program (BRP), King Saud University, Riyadh, KSA. A self-administered questionnaire was given to all participating subjects containing demographic and dietary information, particularly the frequency of coffee and tea intake. No distinction was made between the types of coffee (filtered, boiled, instant, pads) and between caffeinated and decaffeinated coffee. Subjects were divided into three groups based on coffee and tea consumption: Group I included those who drank 0to 4 cups of tea or coffee weekly, group II included those who consume 5–8 cups of tea or coffee per week and group III included those who consume tea or coffee 8–12 times per week. Furthermore, the questionnaire also sought information about sun exposure [frequency of exposure (e.g. no exposure, daily weekly) and physical activity [inactive, moderate, active], which were self reported.

Children with co-morbidities that needed immediate medical attention were excluded from the study. Written consent was obtained after orientation of the study protocol. Ethical approval was granted by the Ethics Committee of the College of Science Research Center, King Saud University, Riyadh, KSA.

### Anthropometry and blood collection

Participating subjects were requested to return to their respective PHCCs after an overnight fast for anthropometry and blood withdrawal. Anthropometry included height (to the nearest 0.5 cm), weight (to the nearest 0.1 kg), utilizing a standardized measuring tape in cm; and BMI (calculated as kg/m^2^). Blood was transferred immediately to a non-heparinized tube for centrifugation. Serum was then transferred to a pre-labeled plain tube, stored in ice, and delivered to the Biomarker Research Program in King Saud University on the same day.

### Sample analyses

Fasting serum samples were stored in a −20C freezer until analysis. Fasting blood glucose, lipid profile, phosphorus and calcium were measured using a chemical analyzer (Konelab, Vantaa, Finland). Serum 25-OH-VitD was measured by enzyme linked immunosorbent assays (ELISA) (IDS Ltd, Boldon Colliery, Tyne & Wear, UK) according to manufacturer's instruction. The inter- and intra-assay variabilities for 25-OH D ELISA were 5.3% and 4.6% respectively. All sample measurements were done in BRP, a participating laboratory for DEQAS (Vitamin D External Quality Assessment Scheme) based in UK.

### Statistical analysis

Data was analyzed using the Statistical Package for the Social Sciences (SPSS for Windows version 16.5). Data are expressed as mean ± standard deviation for normally distributed parameters. Group comparisons were done using analysis of covariance (ANCOVA), adjusted for age and BMI. P-value less than 0.05 was deemed significant.

## Results

Comparisons between subjects based on coffee consumption are presented in Table 1. In both males and females, those consuming the most coffee cups per week were significantly younger with lower BMI, lean and soft body mass as compared to those who were drinking coffee less. In males, LDL- and HDL-cholesterol levels were highest among those consuming coffee 9–12 times week (p-values 0.022 and 0.0008 respectively) and although the same trend were observed in females, only HDL-cholesterol was significant. Circulating 25-hydroxyvitamin D levels were significantly elevated among girls consuming coffee 9–12 times per week, even after adjusting for age and BMI.

**Table 1:** Comparison of male and female subjects based on coffee intake

	**Males**				**Females**			
**Parameters**	**Group 1**	**Group 2**	**Group 3**	**p-value**	**Group 1**	**Group 2**	**Group 3**	**p-value**
	**0–4**	**5–8**	**9–12**		**0–4**	**5–8**	**9–12**	
	**times/week**	**times/week**	**times/week**		**times/week**	**times/week**	**times/week**	
N	60	16	75		83	22	70	
Age(years)	13.2 ± 3.1	13.4 ± 2.5	11.6 ± 3.4	0.009	13.5 ± 3.2	12.0 ± 3.6	12.3 ± 3.3	0.035
BMI (kg/m^2^)	22.6 ± 8.2	22.9 ± 6.8	19.5 ± 5.1	0.020	23.1 ± 7.1	18.6 ± 4.0	21.3 ± 6.4	0.016
Sun Exposure (%) (Daily/weekly)	60/25	31/50	44/36	0.16	20.7/26.5	22.7/50.0	28.5/50.0	0.001
Physical Activity (%)(moderate/Active)	20/46.6	25/62.5	10.6/64.0	0.14	13.2/65.0	22.7/68.1	15.7/67.1	0.39
Waist (cm)	67.4 ± 30.0	72.3 ± 14.6	63.7 ± 15.1	0.343	65.5 ± 23.0	62.4 ± 10.0	68.2 ± 12.4	0.413
Hips (cm)	78.3 ± 30.6	89.3 ± 16.4	77.6 ± 20.1	0.246	82.2 ± 24.4	79.4 ± 13.7	86.4 ±15.4	0.299
LBM (kg)	40.8 ± 15.2	34.2 ± 10.0	32.1 ± 12.8	0.010	34.1 ± 9.2	27.4 ± 8.9	31.3 ± 8.9	0.015
SLM (kg)	36.2 ± 14.3	31.8 ± 9.5	28.9 ± 12.3	0.011	32.0 ± 9.4	25.3 ± 8.4	28.8 ± 8.4	0.010
Glucose (mmol/l)^†^	5.2 ± 0.77	5.4 ± 1.0	5.3 ± 0.60	0.193	5.0 ± 0.85	5.0 ± 0.65	5.1 ± 0.52	0.299
Triglycerides (mmol/l)^†^	0.94 ± 0.05	1.1 ± 0.08	0.80 ± 0.04	0.111	0.94 ± 0.08	0.78 ± 0.02	0.86 ± 0.03	0.243
HDL (mmol/l)^†^	0.89 ± 0.35	1.05 ± 0.20	1.1 ± 0.26*	0.022	1.02 ± 0.31	1.1 ± 0.19	1.2 ± 0.25*	0.034
LDL(mmol/l)^†^	2.9 ± 0.68	2.8 ± 0.58	2.5 ± 0.57*	0.008	2.9 ± 0.66	2.6 ± 0.57	2.7 ± 0.60	0.088
T. Cholesterol(mmol/l)^†^	4.3 ± 1.5	4.2 ± 0.65	3.8 ± 0.62*	0.043	4.3 ± 0.65	3.9 ± 0.56	4.1 ± 0.66	0.061
Serum Ca (mmol/l)^†^	2.5 ± 0.26	2.5 ± 0.16	2.6 ± 0.16*	0.008	2.4 ± 0.19	2.6 ± 0.12	2.5 ± 0.20	0.100
Corrected Ca (mmol/l)^†^	2.6 ± 0.32	2.6 ± 0.39	2.6 ± 0.24	0.582	2.4 ± 0.38	2.5 ± 0.10	2.5 ± 0.20	0.931
Albumin (gm/L)^†^	45.5 ± 4.5	45.8 ± 4.0	45.1 ± 3.2	0.891	44.8 ± 3.4	46.5 ± 2.7	44.3 ± 3.6	0.050
Serum Phosphorus ^†^	1.6 ± 0.63	1.5 ± 0.30	1.6 ± 0.38	0.891	1.4 ± 0.9	1.4 ± 0.24	1.3 ± 0.30	0.481
Vitamin D (nmol/l)^†^	24.0 ± 1.5	20.7 ± 1.3	25.4 ± 1.6	0.212	16.7 ± 1.6	19.4 ± 1.5	20.5 ± 1.5*	0.020

Data represented by Mean ± standard deviation;

‘†’Represents Analysis of Co variance (ANCOVA) is done across the coffee drinkers, Adjusted for age and BMI.

**Figure 1:**
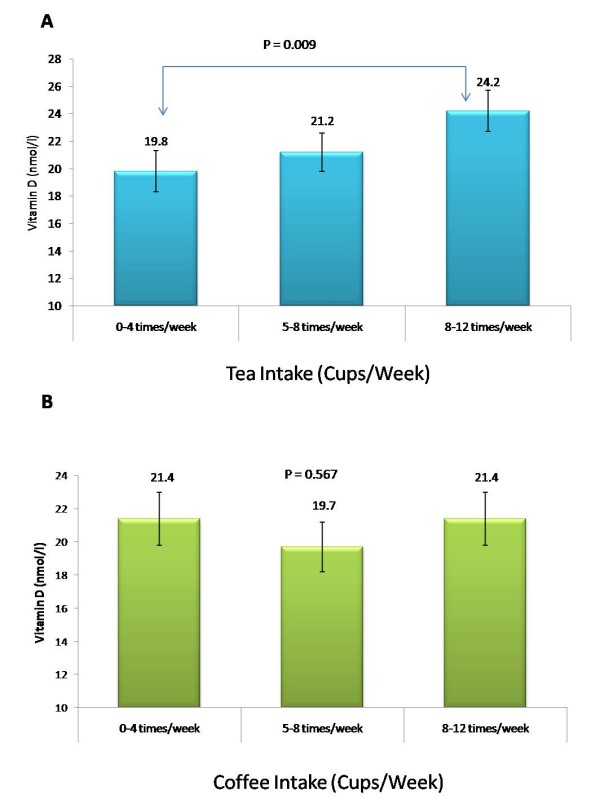
**Levels of 25(OH)D according to tea (A) and coffee (B) consumption.** Comparisons were adjusted for age, BMI, gender, physical activity and sun exposure. Significance set at ***p*** **< 0.05.**

Comparisons between subjects based on tea consumption are presented in Table 2. While most of the variables were comparable with one another in both genders, vitamin D levels were observed to be highest among both genders consuming the most tea per week (9–12 times/week) and these significance was independent from age and BMI. Figure 1 shows the effect of increased tea intake (1A) in the vitamin D status of subjects independent of age, gender, BMI, sun exposure and physical activity. Subjects who consumed 0–4 times of tea per week had significantly lower 25(OH)D levels as compared to those consuming 8–12 times/week. No significant difference in 25(OH)D was found when compared to coffee intake (B).

**Table 2:** Comparison of male and female subjects based on tea intake

	**Tea Intake**							
	**Male**				**Female**			
**Parameters**	**Group 1**	**Group 2**	**Group 3**	**p-value**	**Group 1**	**Group 2**	**Group 3**	**p-value**
	**0–4**	**5–8**	**9–12**		**0–4**	**5–8**	**9–12**	
	**times/week**	**times/week**	**times/week**		**times/week**	**times/week**	**times/week**	
N	96	23	36		105	17	53	
Age(years)	12.7 ± 3.3	12.5 ± 3.2	11.7 ± 3.5	0.274	13.0 ± 3.4	12.5 ± 3.4	12.7 ± 3.2	0.808
BMI (kg/m^2^)	21.1 ± 6.6	23.7 ± 8.0	19.9 ± 6.7	0.177	22.1 ± 6.2	21.2 ± 3.6	21.5 ± 8.0	0.763
Sun Exposure (%) (Daily/weekly)	54/29.1	30.4/30.4	38.8/47.2	0.17	19.0/34.2	23.5/47.0	22.6/41.5	0.06
Physical Activity (%)(moderate/Active)	21.0/51.0	13.0/60.8	11.0/63.8	0.04	16.1/64.7	17.6/76.4	15.1/67.9	0.91
Waist (cm)	65.5 ± 23.6	69.2 ± 24.6	65.7 ± 17.6	0.816	64.2 ± 18.0	67.8 ± 8.8	69.9 ± 18.4	0.177
Hips (cm)	77.4 ± 25.4	83.8 ± 25.9	80.7 ± 22.6	0.555	81.0 ± 22.0	86.8 ± 8.2	87.7 ± 18.1	0.138
LBM (kg)	35.7 ± 13.6	36.7 ± 13.8	33.5 ± 14.8	0.672	32.4 ± 9.7	32.2 ± 6.2	31.6 ± 9.3	0.913
SLM (kg)	32.5 ± 12.9	34.2 ± 13.1	29.9 ± 14.1	0.510	30.4 ± 9.8	29.8 ± 5.8	29.1 ± 8.9	0.738
Glucose (mmol/l)^†^	5.2 ± 0.81	5.3 ± 0.38	5.3 ± 0.69	0.904	4.9 ± 0.78	5.0 ± 0.72	5.2 ± 0.51	0.160
TG (mmol/l)^†^	0.90 ± 0.05	1.1 ± 0.07	0.78 ± 0.04	0.062	0.87 ± 0.04	0.99 ± 0.12	0.90 ± 0.04	0.600
HDL (mmol/l)^†^	1.0 ± 0.34	1.0 ± 0.22	1.01 ± 0.23	0.959	1.0 ± 0.29	1.2 ± 0.23*	1.1 ± 0.24*	0.003
LDL(mmol/l)^†^	2.8 ± 0.61	2.6 ± 0.63	2.4 ± 0.65*	0.042	2.9 ± 0.64	2.7 ± 0.37	2.7 ± 0.68	0.274
Total Cholesterol(mmol/l)^†^	4.2 ± 1.2	3.9 ± 0.67	3.8 ± 0.70	0.111	4.2 ± 0.66	4.1 ± 0.40	4.0 ± 0.71	0.667
Serum Ca (mmol/l)^†^	2.5 ± 0.24	2.6 ± 0.21	2.6 ± 0.20	0.322	2.5 ± 0.20	2.4 ± 0.18	2.6 ± 0.23	0.184
Corrected Ca (mmol/l)^†^	2.6 ± 0.34	2.4 ± 0.20	2.5 ± 0.23	0.132	2.5 ± 0.33	2.5 ± 0.21	2.5 ± 0.17	0.972
Albumin (gm/L)^†^	45.7 ± 3.9	45.7 ± 4.4	44.6 ± 3.2	0.427	44.8 ± 3.9	44.6 ± 2.7	44.6 ± 2.9	0.936
Serum Phosphorus ^†^	1.6 ± 0.56	1.5 ± 0.31	1.5 ± 0.25	0.741	1.4 ± 0.9	1.3 ± 0.2	1.4 ± 0.30	0.802
Vitamin D (nmol/l)^†^	22.5 ± 1.5	23.5 ± 1.4	30.0 ± 1.5*	0.003	17.4 ± 1.6	17.2 ± 1.4	21.2 ± 1.5*	0.031

Data represented by Mean ± standard deviation;

Analysis of Variance (ANOVA) is done across different tea drinkers.

‘*’ represents group is significantly from the group 1.

## Discussion

To the best of our knowledge, there is no recorded study on the association of caffeine intake with circulating levels of 25-hydroxyvitamin D. Current evidence however relate caffeine intake to calcium metabolism [10-12]. It has been demonstrated that caffeine negatively influences calcium balance by reducing renal reabsorption of calcium, and possibly by reducing intestinal calcium absorption efficiency. High caffeine intake may involve considerable renal and intestinal calcium losses [13]. Results of previous epidemiological studies have suggested a relationship between high caffeine consumption and low bone mineral density (BMD) and osteoporotic fractures, which however may be offset by a high calcium intake [14,15]. Caffeine intake also reduces inositol levels in the blood. Inositol is a regulating factor in calcium metabolism [13], and can modestly increase calcium excretion and reduce absorption (Barrett-Connor et al., 1994). Caffeine intake >300 mg/d (≈514 g, or 18 oz, brewed coffee) accelerate bone loss at the spine in elderly postmenopausal women [7]. Furthermore, women who harbor the TT genetic variant of *VDR* appear to be at a greater risk for this deleterious effect of caffeine on bone [7]. The polymorphisms in the VDR gene correlate with BMD, bone turnover and bone loss [16,17]. In our study, serum vitamin D level increases as coffee and tea consumption increases. Increased caffeine dose decreases VDR protein expression and alkaline phosphatase enzyme activity, a marker of osteoblast differentiation in osteoblast cells [18]. Caffeine is also metabolized in the liver via the cytochrome P450 oxidase enzyme system [19,20]. About a dozen metabolites can be recovered in the urine of regular coffee consumers [19,21]. Methylxanthine, theophylline and caffeine were found to inhibit the conversion of 25 hydroxyvitamin D3, to 1,25 dihydroxyvitamin D3 in isolated renal tubules in vitamin D deficient chicks, which led to increased vitamin D circulating levels [22].

The role of caffeine as a risk factor for bone loss is controversial. Moderate coffee consumption has no effect on bone health [13]. However, low calcium intake is clearly linked to skeletal fragility, and it is likely that a high caffeine intake is often a marker for low calcium intake [11]. The negative effect of caffeine on calcium absorption is small enough to be fully offset by as little as 1–2 tablespoons of milk. All of these observations implicating caffeine-containing beverages as a risk factor for osteoporosis have been made in populations consuming substantially less than optimal calcium intakes [11].

Another factor that may contribute to higher vitamin D in the heavy drinkers is the weight reducing effect of coffee. In our study BMI mean value was significantly decreased in heavy drinkers when compared to low and moderate drinkers (mean of BMI =20 ± 5.8 kg/m2, P < 0.05). Both sexes showed a decrease in BMI (appendix). In a study done by Jorde et al. (2001), both gender's serum calcium showed a positive association with body mass index (BMI) and coffee consumption that persisted after correcting for other variables in a multiple regression model (P < 0.05) [23].

Coffee intake should reduce BMI because coffee consumption decreases the amount of fat tissue by elevating thermogenesis [24-26] and stimulating the sympathoadrenal system [27]. Aside from caffeine's ability to increase fat oxidation and lipolysis [28-30], there is a weak but significant positive association with BMI for coffee intake in both sexes [30]. Coffee may reduce body weight through lipolysis stimulation [27]. Fat loss from adipose tissue is hypothesized to be accompanied by vitamin D withdrawal from its fat store leading to elevated serum vitamin D levels. Studies on weight reduction show that serum 25(OH)D levels rise when obese individuals lose body fat [31-33].

There is consistent epidemiological evidence that consumption of these hot beverages is associated with a reduced risk of diabetes mellitus type 2 (DMT2) and coronary heart disease [34-36]. It has been hypothesized that the protective effect of coffee and tea consumption on DMT2 risk is mediated by a reduction in obesity [37]. This hypothesis is supported by a handful of clinical intervention studies which report that the frequency of coffee or tea consumption is related to weight loss and change in body fat distribution [37,38].

The study has limitations. Comprehensive data on outdoor physical activity and diet were lacking, and these can be considered major confounders that affect circulating vitamin D levels. The type of coffee and tea were also not taken into consideration. Nevertheless, the number of subjects involved increases the reliability of our results, which shows for the first time a dose-dependent relationship between circulating vitamin D levels in relation to coffee and tea consumption among Saudi adolescents, even after adjustment for physical activity, sun exposure, gender, age and BMI.

In summary, the study suggests that increased coffee and tea consumption elevates circulating vitamin D levels among Saudi Arab adolescents independent of physical activity, sun exposure, age, gender and BMI. Further studies are needed to confirm these findings as assessment of the health risks and benefits of coffee and tea consumption requires reliable data that can accurately for studying relationships between coffee and tea consumption and health-related endpoints.

## Competing interests

The authors declare that they have no competing interests

## Authors’ contributions

AA, SA and NMA conceived and carried out the study. KMA, YA, OSA and MSA participated in the design, subject recruitments and data collection. OM and SY carried out sample analysis. SA, SS, SK and GPC performed statistical analysis and drafted the final version of the manuscript. All authors approved and read the final manuscript.
